# Quality of Medication Abortion Services From Pharmacies and Drugstores in Ethiopia: A Two‐Stage Study

**DOI:** 10.1111/1471-0528.70110

**Published:** 2025-12-15

**Authors:** Tesfaye Tufa, Stephanie Andrea Küng, Delayehu Bekele, Niguse Tadele, Mahari Yihdego, Kidist Lemma, Alice F. Cartwright

**Affiliations:** ^1^ University of Toronto Toronto Ontario Canada; ^2^ Guttmacher Institute New York City New York USA; ^3^ St. Paul Institute for Reproductive Health and Rights Addis Ababa Ethiopia; ^4^ Addis Ababa University Addis Ababa Ethiopia; ^5^ Performance Monitoring for Action Ethiopia Addis Ababa Ethiopia; ^6^ St. Paul's Hospital Millennium Medical College Addis Ababa Ethiopia

**Keywords:** abortion care, drugstore, medication abortion, mystery client, pharmacies

## Abstract

**Objective:**

To assess the quality of medication abortion (MA) services provided in pharmacies and drugstores (‘pharmaceutical outlets’) in Ethiopia.

**Design:**

A two‐stage cross‐sectional study.

**Setting:**

Pharmaceutical outlets in Addis Ababa, Ethiopia.

**Sample:**

Phase 1: 1696 pharmaceutical outlets listed in the Ethiopian Federal Ministry of Health Master Facility Registry, plus 187 additional outlets identified in the field. Phase 2: Selected 600 pharmaceutical outlets.

**Methods:**

After assessing stock availability in phase 1, during phase 2, mystery clients (MCs) visited pharmaceutical outlets to evaluate service quality using an adapted version of the Abortion Care Quality (ACQ) Tool. Descriptive statistics were used to characterise the quality of services provided by the outlets.

**Main Outcome Measures:**

The quality of abortion services provided by pharmaceutical outlets.

**Results:**

Abortion medications were sold without prescriptions in 23.5% of MC visits. Among these sales, client respect (95.7%) and confidentiality (84.4%) were high. Additionally, 67.1% of staff gave correct instructions on dosage, timing, and administration of the medications. However, less than half of the MCs received adequate information on possible complications (36.4%). Almost all medications purchased were unexpired, packaged in aluminum, and of a known brand (96.5%); however, none were characterised as affordable.

**Conclusions:**

Pharmaceutical outlets demonstrated moderate quality for MA services, yet there were notable gaps in counselling for physical side effects and complications, and affordability challenges. With appropriate policy adjustments and training interventions, there is potential to integrate pharmaceutical outlets into the abortion care service delivery infrastructure, ensuring equitable access to safe and effective abortion services in Ethiopia.

## 1 Introduction

Ethiopia has made notable progress in improving reproductive healthcare access and reducing maternal mortality, from 871 per 100 000 live births in 2000 to 267 in 2020 [1, 2]. This progress can be attributed to several key factors, including the liberalisation of abortion laws in 2005, which led to substantial health reforms such as the training of healthcare workers on abortion care, the expansion of services at various health service levels, and the introduction of medication abortion (MA). These changes paved the way for increased access to quality abortion care services, aligning with the broader global shift toward recognising reproductive rights as essential components of public health [3, 4, 5, 6].

Medication abortion is an important method for terminating pregnancies in Ethiopia, offering a non‐invasive alternative to surgical procedures [3]. Both medications (misoprostol and mifepristone) recommended for use in MA are registered by the Ethiopian Food and Drug Authority (EFDA) [7]. The global trend toward expanding access to MA is supported by evidence indicating that when provided with appropriate guidance, women can safely manage their abortions, reducing the need for clinic‐based procedures and improving access in rural and underserved areas [8, 9, 10].

Pharmaceutical outlets play a vital role in Ethiopia's healthcare system, often serving as the first point of contact for many individuals seeking medical assistance [11, 12]. Pharmaceutical outlets include both pharmacies and drugstores; the main difference between pharmacies and drugstores lies in the staff (licensed pharmacists work at pharmacies vs. licensed drugstore staff working at drugstores) and the broader scope of services at pharmacies. However, the role of pharmaceutical outlets in the management of abortion is restricted to dispensing the medications prescribed by a healthcare provider. According to the Ethiopian Federal Ministry of Health (FMOH) guidelines, information provision, counselling, and eligibility assessment for abortion must be performed in a health facility by a trained healthcare provider [13]. However, anecdotal reports suggest that mifepristone and misoprostol can be procured from pharmaceutical outlets without prescriptions. Some people may prefer to obtain MA from pharmaceutical outlets due to their physical proximity, the potential stigma faced in health facilities, and the perception of increased confidentiality in these outlets [14, 15].

While some research in low‐ and middle‐income countries (LMICs) has highlighted concerns over the quality of abortion care provided in pharmaceutical outlets, other studies have shown the potential of pharmaceutical outlets to manage and expand abortion services effectively [16, 17]. In some cases, these providers may lack adequate training or may not follow best practices for counselling or managing complications [16]. However, the quality of MA services provided in Ethiopian pharmaceutical outlets has not been assessed.

Our study addresses this knowledge gap by using a mystery client (MC) approach, which has been employed in various settings to explore MA service provision in pharmaceutical outlets [17, 18, 19]. This approach simulates real‐life interactions, capturing the actual behaviour of pharmacy and drugstore staff rather than responses influenced by observation or interviews.

## 2 Methods

### 2.1 Study Design

We conducted a two‐stage cross‐sectional study in pharmaceutical outlets in Addis Ababa, Ethiopia, between July and September 2024. The first phase was a stock survey to determine the availability of MA medications, and the second phase was a MC survey to assess service quality and provider practices. This study received approval from the institutional review boards of the Guttmacher Institute and the St. Paul's Hospital Millenium Medical College (SPHMMC).

### 2.2 Sample

In Phase 1, we aimed to collect stock information from all pharmaceutical outlets in Addis Ababa. Phase 2 used a purposive sampling approach for the MC study, visiting all 446 outlets that had reported stocking MA medications (mifepristone and/or misoprostol) in Phase 1, plus an additional 154 outlets that had reported not stocking [proportional to the distribution of pharmaceutical outlets in the FMOH's Master Facility Registry (MFR)], for a total of 600 outlets visited by MCs.

In Ethiopia, medicines must be registered with the Ethiopian Food and Drug Authority to ensure their quality, safety, and efficacy. Only EFDA‐registered products can be legally imported and dispensed, making registration status an important marker of authorised versus informal supply [20].

### 2.3 Data Collection

#### 2.3.1 Phase 1: Stock Survey

The stock survey, conducted in July 2024, documented the availability, storage, and stocking patterns of 21 reproductive and maternal health medications and supplies, including mifepristone and misoprostol. Thirty trained data collectors conducted face‐to‐face interviews using a structured questionnaire. Data collectors were trained to ask for the person in charge; these staff (employees, owners, and managers) were provided with information about the objective of the stock survey (Phase 1) without disclosing information about the MC survey (Phase 2), and oral consent was obtained. Key data points included pharmaceutical outlet characteristics (such as working hours and geographic location), day‐of and three‐month retrospective stock levels, and storage conditions (specifically for MA medications). Interview responses were recorded on encrypted smartphones using Open Data Kit (ODK).

#### 2.3.2 Phase 2: Mystery Client Survey

The MC survey, conducted in August and September 2024, assessed staff practices, service quality, and sales of MA medications from the perspective of simulated clients. MCs were female theatre school graduates or students, recruited from local communities, and aged 19–25 years to align with the demographic profile of women most likely to seek MA services in Addis Ababa [21]. MCs were trained using role‐playing sessions to use standardised scripts when approaching any staff member at the service counter for assistance for a “missed period,” probing for more information from staff regarding dosage, timing of medication, and possible complications, and were given predetermined responses to anticipated provider queries. If asked, MCs were instructed to tell staff that they were students, lived with family, and had a boyfriend. Measures were taken to protect MCs' safety and anonymity, including the use of fictitious names and temporary phone numbers. Mystery clients were trained to disengage from interactions if they felt unsafe.

Mystery clients provided a date of last menstrual period (LMP) only if asked during the visit. In 90% of visits, it was equivalent to a gestational age of 8 weeks, and in 10% of visits, it was equivalent to 14 weeks. This allocation was intended to reduce refusals due to regulatory restrictions on second‐trimester MA services, but to gather exploratory evidence of sales as gestational age advances. Mystery clients were provided with 2000 Ethiopian birr (ETB) (18.15 USD) (average conversion rate at time of data collection [August 2024]) for their purchases. If the price quoted was higher than this amount, MCs were trained to negotiate and, if negotiation was not possible, left the interaction and awaited guidance from the study team. MA was purchased in all cases where offered, regardless of price.

Mystery clients were paired with trained data collectors who conducted exit interviews at a nearby private location immediately after the MCs left the pharmacy or drugstore. The exit interviews captured details about the client‐staff interaction, including whether pharmaceutical outlet staff refused to sell MA and MC‐reported reasons for refusal, the types of medications sold, instructions provided, cost of medications, and other information related to the quality of care provided. Data collectors followed a standardised survey to record MC responses using a smartphone via ODK.

### 2.4 Measures

Quality of care was measured using the Abortion Care Quality Tool (ACQTool) [22]. The ACQTool was originally designed to assess care provided in health facilities, pharmacies, and through hotlines. Given our MC design, we modified the tool. These modifications were made due to data limitations inherent in the MC design—specifically its reliance on simulated client encounters that capture only observable interactions and without the ability to directly question staff about their knowledge, and to ensure stricter criteria for certain indicators. We included 21 of the original 25 ACQTool indicators for quality abortion care from pharmacies and modified seven indicators for our MC study design (Table S1). For example, to pass the emergency referral system indicator, the pharmacy and drugstore staff had to inform the MC about potential complications *and* mention where to go in case of complications. MA medications were considered high quality if they were unexpired, packaged in aluminum, and of a known brand. To determine affordability, we considered the use of multiple thresholds (1) the WHO‐defined medication affordability measure, which uses the minimum daily wage of government employees (2), the daily salary of someone living in Ethiopia at the extreme poverty threshold [23, 24], and (3) a measure assuming a 100% markup of the sales price to pharmacies from medication suppliers, which we gathered through personal communication. Of these, we used the third 300 ETB (2.72 USD), in determining whether a purchase was considered “affordable.” Pharmacy and drugstore staff were considered technically competent if they provided accurate dosage, route, and administration instructions appropriate for gestational age in line with FMOH guidelines [13]. We also checked whether the pharmacy or drugstore staff assessed the MC for contraindications and correctly informed them of side effects and complications (See Table 2 for definitions of technical competencies).

### 2.5 Analysis

We ran descriptive statistics of all variables of interest. Agreement or refusal to sell medications, as well as reasons for refusal, were assessed among the full sample of pharmaceutical outlets from the MC survey (*n* = 600). ACQTool quality of care measures were assessed among MC visits where purchases were made. We used chi‐square tests to assess differences in service provision quality by facility type. All analyses were conducted using Stata 18.0.

### 2.6 Patient and Public Involvement

A Technical Advisory Group (TAG) comprising representatives from the FMOH, Non‐Governmental organizations (NGOs), and teaching hospitals guided the study design and data collection. The TAG ensured alignment with national priorities and ethical standards. Abortion clients were not involved in the study's recruitment or data collection processes.

## 3 Results

### 3.1 Sales and Refusals

**FIGURE 1:**
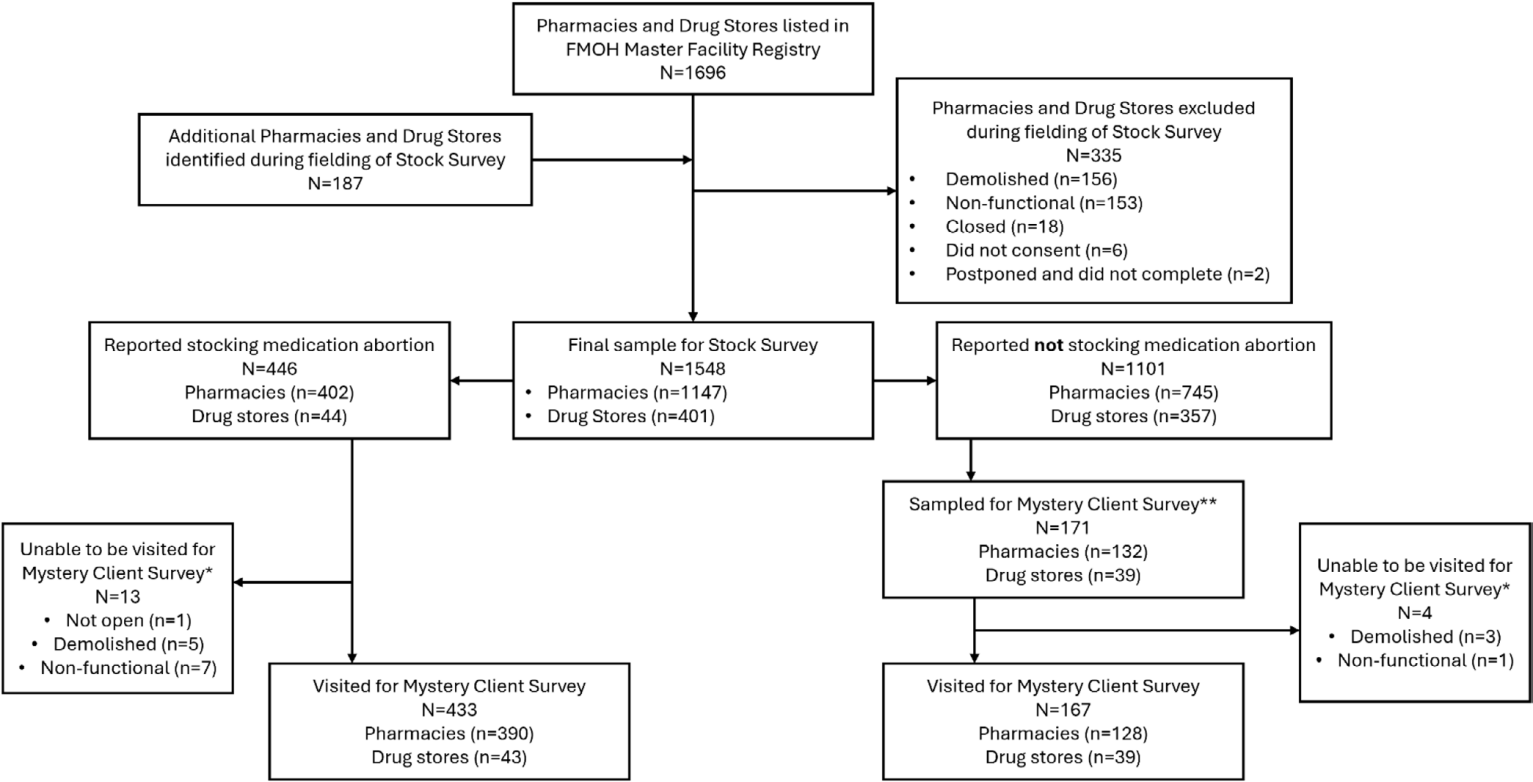
Pharmacies and drug stores visited in Addis Ababa, Ethiopia, and stock survey and mystery client survey samples. *These outlets were replaced in the sample with additional pharmacies/drug stores that do not stock; **Number of pharmacies and drug stores that do not stock sampled was proportional to their distribution in the overall Stock Survey. FMOH = Federal Ministry of Health.

Phase 1 included 1696 pharmacies and drugstores listed in the FMOH's MFR, and an additional 187 outlets identified in the field that were not on the Registry (Figure 1). We ultimately collected data from 1548 outlets (82.3% response rate).

**FIGURE 2:**
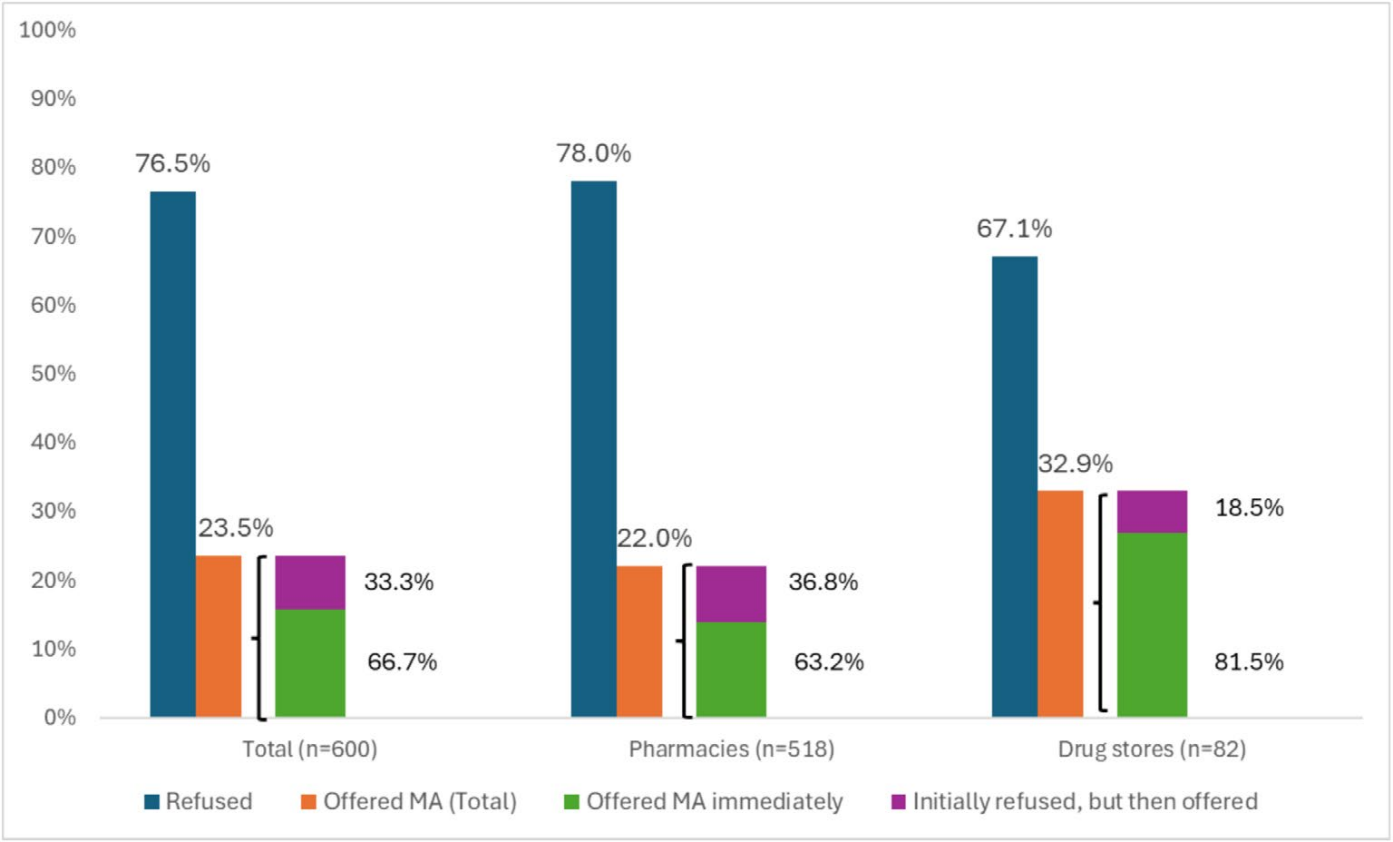
Refusals and purchases of abortion medications by mystery clients at pharmacies and drug stores in Addis Ababa, Ethiopia (*n* = 600). MA = medication abortion.

Mystery clients completed 600 visits to 518 pharmacies and 82 drugstores in all 11 sub‐cities in Addis Ababa. MCs were able to purchase MA medications without a prescription in nearly one‐quarter (23.5%, *n* = 141) of visited pharmaceutical outlets, with a success rate of 22.0% for pharmacies and 32.9% for drugstores (Figure 2). Only 15.0% of 14‐week gestation MCs (*n* = 6) were able to purchase abortion medications (data not shown). The main reasons for outright refusal included the pharmaceutical outlet not selling abortion medications (60.6%) or the MC not having a prescription (28.8%). Of the successful purchases, two‐thirds (66.7%) were offered abortion medications immediately, while 33.3% were initially refused but ultimately offered. Among the 141 MC visits in which a purchase was made, nearly all (140; 99.3%) were sold mifepristone and misoprostol co‐packaged in combi‐packs. One MC was sold oral contraception for termination of pregnancy.

### 3.2 Quality of Care: Stock, Client‐Provider Interactions, and Affordability

We assessed medication and equipment and supply availability as defined by the ACQTool. Less than 15% of drugstores where purchases were made had continuous stock of abortion medications in the 3 months preceding the stock survey, compared to 41.2% of pharmacies (Table 1). Almost all the abortion medications purchased were unexpired, packaged in aluminum, and of a known brand (medication quality: 96.5%). Menstrual pads were continuously in stock for the past 3 months at 84.4% of outlets.

**TABLE 1:** Percentage of pharmacy and drugstore interactions meeting quality care indicators, among mystery clients sold something for a missed period (*n* = 141).

	% mystery clients who made a purchase endorsing quality indicator		
	TOTAL (*n* = 141)	By facility type, with 95% CIs	
		Pharmacies (*n* = 114)	Drugstores (*n* = 27)
Referral systems			
Emergency referral systems^b^ (*n* = 138)	31.2	33.0 (24.2, 41.8)	23.1 (6.1, 40.1)
Supplies, medicines, and equipment			
Equipment and supply availability^c^	84.4	84.2 (77.4, 91.0)	85.2 (71.1, 99.2)
Medication availability^a^, ^c^	36.2	41.2 (32.1, 50.4)^a^	14.8 (0.8, 28.9)^a^
Client perception of medication quality^b^	96.5	96.5 (93.1, 99.9)	96.3 (88.8, 100.0)
Access			
Affordability^b^ (*n* = 140)	0.0	0.0	0.0
Hours of operation (*n* = 600)	99.7	99.6 (99.1, 100.0)	100.0
Service refusal^a^, ^b^ (*n* = 600)	76.5	78.0 (74.4, 81.6)	67.1 (56.7, 77.4)
Technical competence			
Pain management^b^	60.3	63.2 (54.2, 72.1)	48.1 (28.4, 67.9)
*Medication abortion procedure competence* ^b^ (*see* Table 2)			
Client perception of safety	70.2	67.5 (58.9, 76.2)	81.5 (66.1, 96.8)
Decision making			
No contraceptive method coercion	89.4	90.4 (84.9, 95.8)	85.2 (71.1, 99.2)
Personalised care options	44.7	45.6 (36.4, 54.9)	40.7 (21.3, 60.2)
Provider support for client decision	80.9	79.8 (72.4, 87.3)	85.2 (71.1, 99.2)
Information provision			
Client communication comfort	80.9	79.8 (72.4, 87.3)	85.2 (71.1, 99.2)
Sufficient provider explanation	56.7	53.5 (44.3, 62.8)	70.4 (52.3, 88.4)
Prepared if complication occurs	30.5	30.7 (22.1, 39.3)	29.6 (11.6, 47.7)
Can determine complete abortion	47.5	47.4 (38.1, 56.6)	48.1 (28.4, 67.9)
All questions answered	41.8	41.2 (32.1, 50.4)	44.4 (24.8, 64.1)
Client understanding checked	66.7	63.2 (54.2, 72.1)	81.5 (66.1, 96.8)
Client‐provider interactions			
Confidentiality^b^	84.4	85.1 (78.5, 91.7)	81.5 (66.1, 96.8)
Respect	95.7	95.6 (91.8, 99.4)	96.3 (88.8, 100.0)

*Note:* Indicators from pharmacy ACQTool not measured: desired contraceptive method received; provider contraception quotas; client understands process; comfortable sharing information.

^a^ Significant at 5% level of significance.

^b^ Adapted from the ACQTool for mystery client context.

^c^ Data drawn from stock survey results.

Pharmaceutical outlets scored highly on client‐staff interaction quality indicators: 95.7% of MCs felt respected and 84.4% felt their confidentiality was protected (Table 1). During most visits, MCs reported no contraceptive coercion (89.4%), and felt comfortable expressing their needs, questions, and fears (client communication comfort: 80.9%). However, fewer pharmaceutical outlets met quality indicators related to complications: during only 30.5% of visits did MCs feel prepared for potential complications, and in 31.2% of visits, they were informed about where to seek emergency care. Among visits where MCs were informed about complications and told where to go in the case of complications (*n* = 54), staff most frequently told MCs to return to the same outlet (24.1% of visits) or go to any health facility without specification (20.4% of visits; data not shown). Additionally, in fewer than half of visits did MCs report feeling all their questions were answered (41.8%) or that they could determine if an abortion was complete (47.5%). In visits where MCs were told how to determine complete abortion (*n* = 67), most MCs were told the products of conception would be expelled (68.7%; data not shown). No purchases were considered affordable; the lowest price for which MA was sold was 315 ETB in one visit (2.86 USD) and the highest price was 3500 ETB (31.77 USD). Overall, drugstores performed similarly to pharmacies on most quality indicators. There were no significant differences by type of facility, with the exceptions of medication availability and service refusal.

### 3.3 Quality of Care: Technical Competence

In the multi‐part assessment of MA procedure competence (Table 2), 67.1% of pharmaceutical outlets in our sample provided correct information on the dosage, timing, and route of administration for abortion medications. When these aspects were assessed individually, over 82.0% of outlets provided correct instructions on *either* dosage, timing, or route. In visits where abortion medications were sold, gestational age was assessed in over two‐thirds of visits (70.0%), and 82.1% of MCs were asked whether they had taken a pregnancy test. However, our results also show that MCs were asked other screening questions not clinically relevant to the MA service, including their marital status (34.3%) and the reason for wanting the abortion (32.1%). Only a small number of visits (2.9%) involved staff screening for relevant contraindications.

**TABLE 2:** Percentage of pharmacies and drugstores meeting medication abortion (MA) procedure competence indicators, among mystery clients selling MA medications (*n* = 140).

	% mystery clients endorsing quality indicator		
	TOTAL (*n* = 140)	By facility type, with 95% CIs	
		Pharmacies (*n* = 113)	Drugstores (*n* = 27)
Provided WHO/MOH recommended dose, route, timing of MA drugs^a^	67.1	68.1 (59.5, 76.8)	63.0 (43.9, 82.1)
Correct dosage	85.0	85.0 (78.3, 91.6)	85.2 (71.1, 99.2)
Correct route	82.1	84.1 (77.2, 90.9)	74.1 (56.7, 91.4)
Correct timing	84.3	83.2 (76.2, 90.2)	88.9 (76.5, 100.0)
Assessed gestational age (GA) using at least one accepted method^b^	70.0	68.1 (59.5, 76.8)	77.8 (61.3, 94.2)
Screening questions			
Pregnancy test	82.1	85.0 (78.3, 91.6)	70.4 (52.3, 88.4)
Marital status	34.3	32.7 (24.0, 41.5)	40.7 (21.3, 60.2)
Reason for wanting abortion	32.1	34.5 (25.7, 43.4)	22.2 (5.8, 38.7)
Contraindications: at least one correct contraindication assessed^c^	2.9	1.8 (0.0, 4.2)	7.4 (0.0, 17.8)
Side effects: At least one correct side effect mentioned^d^	94.3	94.7 (90.5, 98.9)	92.6 (82.2, 100.0)
Bleeding for a few days	85.7	85.0 (78.3, 91.6)	88.9 (76.5, 100.0)
Abdominal cramping for a few days	65.0	62.8 (53.8, 71.8)	74.1 (56.7, 91.4)
Diarrhoea	4.3	4.4 (0.6, 8.3)	3.7 (0.0, 11.2)
Mild fever or shivering	17.9	19.5 (12.1, 26.8)	11.1 (0.0, 23.5)
Nausea or vomiting	12.9	11.5 (5.6, 17.5)	18.5 (0.0, 33.9)
Complications: At least one correct complication mentioned^e^	36.4	37.2 (28.2, 46.2)	33.3 (14.7, 52.0)
Heavy bleeding^f^	27.1	29.2 (20.7, 37.7)	18.5 (3.2, 33.9)
Bleeding for more than 2 weeks	14.3	14.2 (7.7, 20.7)	14.8 (0.8, 28.9)
Severe pain	6.4	6.2 (1.7, 10.7)	7.4 (0.0, 17.8)
High fever lasting more than 24 h	3.6	3.5 (0.1, 7.0)	3.7 (0.0, 11.2)
Foul smelling discharge	1.4	1.8 (0.0, 4.2)	0.0
Continued pregnancy symptoms	0.7	0.0	3.7 (0.0, 11.2)

^a^ For the first trimester, this is defined as instructing the mystery client (MC) to take 1 mifepristone pill orally, followed by 4 misoprostol pills taken together 24–48 h after mifepristone, either vaginally, buccally, or sublingually. For the second trimester, this is defined as instructing the MC to take 1 mifepristone pill orally, followed by 2 misoprostol pills taken together 24–48 h after mifepristone or until expulsion, either vaginally, buccally, or sublingually.

^b^ Accepted methods of GA screening are asking about the last menstrual period (LMP) or using a gestational age calculator.

^c^ Correct contraindications are: current use of an IUD, allergy to misoprostol, or lower abdominal pain.

^d^ Defined as mentioning at least one correct physical side effect (bleeding, cramping, diarrhoea, mild fever, nausea).

^e^ Defined as mentioning at least one correct complication (heavy bleeding, severe cramping, bleeding for longer than 2 weeks, high fever, foul‐smelling discharge, or continued pregnancy symptoms).

^f^ Defined as soaking more than two sanitary pads per hour for 2 h or bleeding lasting longer than 2 weeks.

Overall, in a majority of visits, pharmaceutical outlet staff correctly mentioned at least one expected physical effect of the abortion medications (94.3%), with bleeding (85.7%) and abdominal cramping (65%) being the most frequently mentioned. Just over one‐third (36.4%) of MCs were correctly informed about at least one potential complication, with heavy bleeding mentioned in one‐quarter of visits (27.1%) in which a purchase was made.

## 4 Discussion

### 4.1 Main Findings

Even though abortion care has become more available and accessible at health facilities in Ethiopia since 2005 [25], this study revealed that abortion medications were able to be purchased without a prescription in approximately one‐quarter of pharmacy and drugstore visits by mystery clients in Addis Ababa, despite restrictions on the sale of these medications without a prescription. While there was a high rate of refusals, for those outlets that did sell the medications, the quality of care provided by staff was reasonably high. Although there are still areas of improvement, this study provides some evidence that MA has the potential to be provided by pharmaceutical outlets, considering that 24% of visits resulted in over‐the‐counter sales. This rate is comparable to similar studies in Nepal (35.7%) and Kenya (28.5%) [17, 26], where sales without a prescription were similarly prohibited. However, the rate of refusals was still high in this study, and the main reason pharmaceutical outlets gave for refusal was that they did not stock the medications or they needed to see a prescription. Consistent stock of abortion medications is a precondition for providing care, and the poor stock continuity of abortion medications, especially in drugstores, underscores the need for supply chain improvements across all service points.

This study is one of the first to use the ACQTool in the assessment of the quality of abortion care in pharmacies, and we found that the quality of care was high across many domains, most notably client‐staff interactions, including respect and confidentiality. This finding is particularly important given the documented challenges and complexities of getting abortion care at health facilities in Ethiopia [14, 27]. Accessing abortion medications through pharmaceutical outlets may be a way for women to avoid experiencing stigma from providers or the risk of being seen by people they know at health facilities [28]. However, respectful care was not universal, so there is still room for improvement, especially considering that the World Health Organization (WHO) emphasises that abortion care should be provided respectfully and compassionately, respecting privacy and confidentiality [29].

Additionally, an essential component of high‐quality abortion care is that people are provided with correct medications and information about how to use them. Encouragingly, only one of the completed sales was for anything other than a mifepristone‐misoprostol combi‐pack. This is likely an effect of Ethiopia's legal environment and that both medications are registered and used for abortion in the country [7]. Interestingly, while over 80% of providers gave the correct information on FMOH guidelines for dosage, timing, *or* route of administration at sale visits, just over two‐thirds provided the correct information for all three. These results indicate there is still a need for improvement but are higher than reported levels of knowledge from pharmaceutical staff in other LMICs [30]. Additional areas for specific improvement included providing sufficient information about the physical effects of MA, warning signs of potential complications, and where to seek emergency care if needed. It is possible that this information was not provided to MCs because staff lacked knowledge, or they feared they would be linked to selling medications without a prescription if a complication did occur. Additionally, the assessment of contraindications was reported to be extremely low. Although over two‐thirds of outlets that sold MA asked about gestational age, very few outlets inquired about relevant medical history that could identify contraindications such as ectopic pregnancy. While the overall risks of MA are low, especially in the first trimester [31], completing these assessments is essential for safe, full‐spectrum abortion care. These are key future areas for additional training among pharmacy and drugstore staff.

One concerning result was related to affordability. Even though women in previous studies, including in Ethiopia, reported choosing to purchase from pharmacies because it was more affordable [28], we did not find that to be the case in this study, with none of the abortion medication sales meeting affordability standards based on several criteria. While it is positive that almost all combi‐pack sales were of unexpired, appropriately packaged, and known brand medications, staff charged high amounts for the medications when they did sell them. These prices are stark when considering that abortion care can be obtained for no cost in public health facilities in Ethiopia [7].

Pharmaceutical outlets already play a substantial role in the provision of SRH services globally, including access to oral contraceptives, emergency contraception, HIV self‐testing kits, and management of sexually transmitted infections. These experiences demonstrate that pharmaceutical outlet‐based SRH services are feasible, widely accepted, and often improve access for underserved populations. Translating this model to MA provision could therefore further expand equitable access, provided quality assurance mechanisms are in place. Pilot efforts to train pharmacy staff to provide MA services directly to patients in the Oromia region, Ethiopia, have already begun to show promise in increasing access to safe and accessible abortion care [32]; this study adds to the increasing evidence base that pharmaceutical outlets could be incorporated into the framework of abortion provision in the country.

### 4.2 Strengths and Limitations

The study demonstrates several strengths. It utilised a two‐stage design, combining stock surveys and MC visits, providing a comprehensive assessment of both stock availability and quality of MA services. The use of the validated ACQTool ensured a robust and standardised evaluation of service quality, facilitating comparisons across settings. Additionally, we ensured that MCs reflected the demographic profile of likely abortion clients and provided them with extensive training. Focusing on Addis Ababa provided valuable insights into service dynamics in a densely populated urban area.

However, the study has limitations. Conducted solely in Addis Ababa, the findings may not be generalizable to rural or other urban regions of Ethiopia. Additionally, because the mystery client study was only implemented in outlets previously identified to stock MA medications (plus a sample of outlets without stock), our results are not necessarily representative of the provision of MA services in *all* outlets in Addis Ababa. We sought to reduce the potential priming of outlet staff to the MC visits from the Phase 1 stock survey by asking about the stock status of a variety of maternal and reproductive health medications; however, there was still a small risk of staff behaviour being influenced if they suspected that the MC request for MA was connected to the previous stock survey data collection. While effective for evaluating service provision, the MC approach may not fully replicate the experiences of actual abortion clients. In addition, because MC studies inherently involve some level of unannounced observation, we did not conduct a post‐visit debriefing with outlets. To minimise any concerns, no outlet identifiers were collected or reported, and findings are shared only in aggregate. Adaptations made to the ACQTool for the MC context limit direct comparability with studies using the original tool. Additionally, we were unable to systematically compare the costs of abortion medications sold without prescriptions to those sold *with* prescriptions, leaving affordability comparisons incomplete. Finally, we provided MCs with up to 2000 ETB to purchase abortion medications, which may have resulted in an overestimation of the rate at which actual clients might be able to purchase. However, MCs were trained to negotiate with staff and the average price paid was less than the maximum amount provided.

### 4.3 Interpretation of the Findings

These findings suggest that pharmaceutical outlets can provide reasonable, moderate‐quality MA services, with notable gaps remaining in counselling on physical side effects, complications, and contraindications, as well as in affordability. Consistent supply chains, staff training, and policy support are essential for improving service delivery in these outlets. Drawing on global evidence that pharmaceutical outlets already play a substantial role in SRH services, expanding their role to include MA services could reduce stigma and improve access.

## 5 Conclusions

In conclusion, this study highlights the critical role pharmaceutical outlets could play in providing MA services in Addis Ababa, Ethiopia. Although pharmaceutical outlets in Ethiopia are not currently permitted to provide abortion medications without a prescription, our research demonestrated that this practice is happening, and several domains of service delivery were performed well. Mystery clients reported respectful treatment, maintenance of confidentiality, and provision of basic regimen instructions, all of which reflect the strength in client interaction and foundational counselling. However, counselling on other components of MA services, such as counselling on potential complications or where to seek emergency care, was inconsistent. Affordability emerged as a major barrier, with substantial variation in price across the pharmaceutical outlets. Addressing these outstanding gaps regarding knowledge, counselling, and affordability is essential to ensuring equitable access to abortion care in Addis Ababa. These findings underscore the potential for the integration of pharmaceutical outlets into the abortion care infrastructure. Targeted training programs could improve technical competence and client support among pharmacy and drugstore staff, and policy reforms are also necessary to enhance drug supply continuity and address affordability challenges. By addressing these issues, Ethiopia can further improve equitable access to safe and effective abortion care, contributing to the broader goal of reducing maternal mortality and morbidity.

## Author Contributions

T.T. conceived the research idea. T.T., S.K., D.B., N.T., M.Y., K.L., and A.F.C. contributed to all aspects of the study, including study design, analysis, and manuscript write‐up. All authors have approved the final version of the manuscript.

## Funding

This project was undertaken with financial support from the Government of Canada, provided through Global Affairs Canada.

## Ethics Statement

Ethical clearance for the research was received from the Institutional Review Boards of St. Paul's Hospital Millennium Medical College (Reference number: PM 23/1062) and the Guttmacher Institute (DHHS identifier IRB00002197).

## Consent

The authors have nothing to report.

## Conflicts of Interest

The authors declare no conflicts of interest.

## Disclaimer

The findings and conclusions in this study are those of the authors and do not necessarily represent the official position of the Guttmacher Institute, St. Paul Institute for Reproductive Health and Rights, University of Ottawa, Addis Ababa University, and Performance Monitoring for Action (PMA) Ethiopia.

## Supporting information


**Table S1:** Adapted ACQ tool indicators used for assessing the quality of care in pharmacies and drug stores.

## Data Availability

The data that support the findings of this study are available on reasonable request from the corresponding author. The data are not publicly available due to privacy or ethical restrictions.
