# Supplementary material for: Quality of Medication Abortion Services From Pharmacies and Drugstores in Ethiopia: A Two‐Stage Study
Source: BJOG. 2025 Dec 15;133(4):796–804. doi: 10.1111/1471-0528.70110 (PMC12884203; doi:10.1111/1471-0528.70110)
Supplement: Supplementary file 1 — Table S1: Adapted ACQ tool indicators used for assessing the quality of care in pharmacies and drug stores. [file BJO-133-796-s001.docx]

**Supplementary Table 1**

| **Domain** | **Measure** | **Used Original ACQTool Indicator** | **Used Adapted ACQTool Indicator** | **Indicator** | **Adapted Indicator** | **Source** |
| --- | --- | --- | --- | --- | --- | --- |
| Referral systems | Emergency referral systems |  | X | Do you have a system for emergency referrals for abortion cases?  What is the emergency referral system? | Did the pharmacist tell you that it is possible you may need follow-up treatment?  Did the pharmacist tell you where to seek help for physical symptoms that suggest there may be a serious problem? | MC Survey |
| Supplies, Medicines, and Equipment | Equipment and Supply Availability | X |  | Check stock card or other stock measurement tool, if available for continuous supply of menstrual pads. | N/A | Stock Survey |
|  | Medication Availability | X |  | Check stock card or other stock measurement tool, if available for continuous supply of mifepristone/misoprostol.  Observe whether MA is well-stored (off the floor, protected from water/sun, no evidence of rodents or pests, storage room well-ventilated, medicines in original packaging), unexpired and packaged in aluminum | N/A | Stock Survey |
|  | Client Perception of Medication Quality |  | X | Do you believe the provider gave you quality medications from a reliable source? | Expiration date of medication? Is the medication packaged in aluminum? Brand name of medication? | MC Survey |
| Access | Affordability |  | X | Do you think the amount you paid for abortion care was affordable? | What was the price of full dose of medication the pharmacist recommended you buy to bring back your period? | MC Survey |
|  | Hours of Operation | X |  | Was it clear to you what hours the pharmacy was open and when you could come in for services? | N/A | MC Survey |
|  | Service Refusal |  | X | List of 10 reasons for which pharmacies reported refusing a client for abortion services in past 12 months. | Did the pharmacy and drugstore staff offer to sell you something to bring your period back? | MC Survey |
| Technical Competence | Pain Management |  | X | Did you feel that your pain was managed effectively? | Did the provider/pharmacist discuss/tell you about ways to manage pain? | MC Survey |
|  | MA Procedure Competence |  | X | List of 12 true/false statements assessing pharmacists’ knowledge of contraindications, medication dosing, and signs of a successful abortion. | Please respond to the following questions based on the instructions that the pharmacist gave you on how to take the MA medication (6 questions to capture dosage, timing, and route instructions) | MC Survey |
|  | Client Perception of Safety | X |  | Did you feel reassured throughout the abortion process that the procedure was safe? | N/A | MC Survey |
| Decision Making | No Contraceptive Method Coercion | X |  | Did the provider make you feel pressured to use a contraceptive method after abortion? | N/A | MC Survey |
|  | Desired Contraceptive Method Received | **Not measured** | | Some people choose to use contraception soon after an abortion, others decide to wait to start a method, and others may decide not to use a method of contraception. Did you want a contraceptive method from this pharmacy? (followed by subsequent questions about method fulfillment or coercion depending on preference) | N/A | N/A |
|  | Provider Contraception Quotas | **Not measured** | | Do you have any target number or quotas for the number of contraceptive methods you are told to provide?  Do you receive any incentive or special privileges for providing higher numbers of contraceptive methods, or certain types of methods? | N/A | N/A |
|  | Personalized Care Options | X |  | Did the provider consider your personal circumstances (including religious beliefs) when discussing procedures with you? | N/A | MC Survey |
|  | Provider Support for Client Decision | X |  | How supportive did you feel that the provider was towards your decision to have an abortion today?  Did the provider or any other staff try to change your mind in any way about having a procedure today, either to convince you to have a procedure or not to have one? | N/A | MC Survey |
| Information Provision | Client Communication Comfort | X |  | Did you feel comfortable expressing your needs, questions, and fears to your provider if you wanted to? | N/A | MC Survey |
|  | Client Understands Process | **Not measured** | | Did the provider explain what was happening during each step of the visit? | N/A |  |
|  | Sufficient Provider Explanation | X |  | Did the provider spend enough time explaining what would happen during each step of the abortion process? | N/A | MC Survey |
|  | Prepared if Complication Occurs | X |  | Do you feel that you know what to do if you experience a warning sign of a complication? | N/A | MC Survey |
|  | Can Determine Complete Abortion | X |  | Did the provider explain to you how to know that your abortion is complete? | N/A | MC Survey |
|  | All Questions Answered | X |  | Did the provider ask if you had questions about what will happen before, during, and after the abortion process, and take the time to answer them? | N/A | MC Survey |
|  | Client Understanding Checked | X |  | Did the provider check with you to make sure that you understood all the information provided? | N/A | MC Survey |
| Client-Provider Interactions | Comfortable Sharing Information | **Not measured** | | Did you feel comfortable sharing personal information with the provider and staff? | N/A | N/A |
|  | Confidentiality |  | X | Do you trust the provider to keep your personal information private? | Did your conversation with the pharmacist take place in a private area, out of hearing range from any other patients or clients? | MC Survey |
|  | Respect | X |  | Do you feel that everyone you spoke with treated you with dignity and respect at all times? | N/A | MC Survey |

**Notes:** ACQTool=Abortion Care Quality Tool, MA=Medication abortion, MC=Mystery client, N/A=Not applicable
